# Supplementary material for: Soluble organic matter Molecular atlas of Ryugu reveals cold hydrothermalism on C-type asteroid parent body
Source: Nat Commun. 2023 Oct 16;14:6525. doi: 10.1038/s41467-023-42075-y (PMC10579312; doi:10.1038/s41467-023-42075-y)
Supplement: Supplementary file 1 — Suplementary Information [file 41467_2023_42075_MOESM1_ESM.pdf]

Soluble organic matter Molecular atlas of Ryugu reveals cold hydrothermalism on C-type asteroid parent body

Philippe Schmitt-Kopplin[1,2,3§], Norbert Hertkorn[2], Mourad Harir[2], Franco Moritz[2], Marianna Lucio[2], Lydie Bonal[4], Eric Quirico[4], Yoshinori Takano[5], Jason P. Dworkin[6], Hiroshi Naraoka[7], Shogo Tachibana[8,9], Tomoki Nakamura[10], Takaaki Noguchi[11], Ryuji Okazaki[7], Hikaru Yabuta[12], Hisayoshi Yurimoto[13], Kanako Sakamoto[9], Toru Yada[9], Masahiro Nishimura[9], Aiko Nakato[9], Akiko Miyazaki[9], Kasumi Yogata[9], Masanao Abe[9], Tomohiro Usui[9], Makoto Yoshikawa[9], Takanao Saiki[9], Satoshi Tanaka[9], Fuyuto Terui[9], Satoru Nakazawa[9], Tatsuaki Okada[9], Seiichiro Watanabe[14], Yuichi Tsuda[9], **Hayabusa2-initial-analysis SOM team***

*** A list of authors and their affiliations appears at the end of the paper**

[§]Corresponding author. Email: schmitt-kopplin@tum.de

Affiliation:

[1] Technische Universität München, Analytische Lebensmittel Chemie; Maximus-von-Forum 2, 85354 Freising, Germany

[2] Helmholtz Munich, Analytical BioGeoChemistry; Ingolstaedter Landstraße 1, 85764 Neuherberg, Germany.

[3]Max Planck Institute for Extraterrestrial Physics, Gießebachstraße 1, 85748 Garching bei München, Germany.

[4]Université Grenoble Alpes, CNRS, CNES, IPAG; 38000 Grenoble, France.

[5]Biogeochemistry Research Center (BGC), Japan Agency for Marine-Earth Science and Technology (JAMSTEC), 2-15 Natsushima, Yokosuka 237-0061, Japan.

[6]Solar System Exploration Division, NASA Goddard Space Flight Center, Greenbelt, Maryland 20771, USA.

[7]Department of Earth and Planetary Sciences, Kyushu University; Motooka 744, Nishiku, Fukuoka 819-0395, Japan.

[8]Tokyo Organization for Planetary and Space Science, University of Tokyo, Bunkyo-ku, Tokyo 113-0033, Japan.

[9]Institute of Space and Astronautical Science, Japan Aerospace Exploration Agency (ISAS/JAXA), Sagamihara 252-5210, Japan.

[10]Department of Earth Material Science, Tohoku University, Aoba-ku, Sendai 980-8578, Japan.

[11]Division of Earth and Planetary Sciences, Kyoto University, Kyoto 606-8502, Japan.

[12]Department of Earth and Planetary Sciences, Hiroshima University, Higashi-Hiroshima, Hiroshima 739-8526, Japan.

[13]Department of Earth and Planetary Sciences, Hokkaido University, Kita-ku, Sapporo 060-0810, Japan.

[14]Graduate School of Environment Studies, Nagoya University, Nagoya 464-8601, Japan.

Supplementary Materials

Supplementary Text

Progressive oxygenation of aliphatic CHO molecules increases the diversity of mid-range atomic environments of carbon atoms up to four bonds away, in particular for remotely oxygenated units ($OC_nC\underline{\mathbf{H}}$; n: 1-2). This structural diversity grows much faster than the nominal O/C elemental ratio. Proportions of remotely oxygenated aliphatic units (OCC**H** units) were larger in Murchison, and directly oxygenated aliphatic (OC**H**) units were distinct in Murchison and A0106 methanol extracts (Figure S8A-D). Remarkably, the A0106 methanol extract showed higher apparent diversity and relative abundance of directly oxygenated aliphatic (OC**H**) units than Murchison extract. When considering rubble-pile asteroid Ryugu as an extinct comet that has lost its icy components (*40*), elevated abundance of oxygenated aliphatic groups and molecules in the methanol extract of a surface sample like A0106 becomes very plausible. Limited bandwidth of overall chemical shift δ_H in ^{1}H NMR spectra decreases opportunities for distinction of alkyl binding motifs in complex mixtures, but variance in curvature of ^{1}H NMR resonances was substantial, reflecting individual distributions of aliphatic branching motifs in Murchison and A0106 methanolic extracts.

Overall, the molecular diversity of aliphatic and aromatic atomic environments with remotely oxygenated carbon atoms was higher in Murchison than in the methanol A0106 extract. Both findings agree with a larger proportion of carboxylic groups, and higher relative abundance of alicyclic binding motifs in Murchison extract. These features decrease the average number of chemical bonds between oxygen and carbon atoms in CHO molecules and induce downfield displacement and line broadening of δ_H for remotely oxygenated aliphatic protons as observed in Murchison extract. Metal complexation through oxygenated functional groups may further contribute to faster relaxation and line broadening in Murchison extract. Enhanced aqueous alteration is a likely cause for oxygenation of organic molecules as well as mobilization of metal ions in asteroid parent bodies.

References Suplementary Meterials:

40. H. Miura, E. Nakamura, T. Kunihiro. The Asteroid 162173 Ryugu: a Cometary Origin. *The Astrophysical Journal Letters.* **925,** L15 (2022).

Table S1: Meteorites and their characteristics used for the model computation (Fig. 4).

Name	Type	Origin	Weathering Grade	Water alteration (*37*) (Rubin et al. 2007)	Petrological type (*38*) (Alexander et al 2013)	Heating stage (*22*) (Quirico et al 2018)	Dataset
**Boriskino**	**CM2**	Fall	0				used for model
**DOM 08003**	**CM2**	Antarctica	2		1.1	TI	used for model
**EET 92042**	**CR2**	Antarctica	2		2.5	TI	used for model
**Essebi**	**C2-ung**	Fall	0		1.9	TI (?)	used for model
**Flensburg**	**C1-ung**	Fall	0	2			used for model
**GRA 95229**	**CR2**	Antarctica	1		2.5	TI (?)	used for model
**GRO 95577**	**CR1**	Antarctica	2	2		TI	used for model
**Ivuna**	**CI1**	Fall	0	1	1	TI	used for model
**LEW 85311**	**CM2_an**	Antarctica	2		1.9	TI	used for model
**LEW 85312**	**CM2_an**	Antarctica	2		1.8	TI	used for model
**MCY 05230**	**CM2**	Antarctica	2		1.8	TI	used for model
**MET 00426**	**CR2**	Antarctica	2		2.6	TI	used for model
**MET 01070**	**CM1**	Antarctica	2	2	1.2	TI	used for model
**Moapa Valley**	**CM1**	Hot desert					used for model
**Mukundpura**	**CM2**	Fall	0			TI	used for model
**Murchison**	**CM2**	Fall	0	2.5	1.6	TI	used for model
**Nogoya**	**CM2**	Fall	0	2.2	1.1-1.6	TI	used for model
**NWA 10923**	**C1_2 ung**	Hot desert	1				used for model
**NWA 8534**	**CM1/2**	Hot desert	1				used for model
**Orgueil**	**CI1**	Fall	0	1	1	TI	used for model
**Paris**	**CM2**	Fall	0	2.9	2.7	TI	used for model
**QUE 99177**	**CR2**	Antarctica	2		2.4	TI	used for model
**Tagish Lake**	**C2_ung**	Fall	0			TI to TII	used for model
**Y791198**	**CM2**	Antarctica		2.4	1.5	TI	used for model
**Yamato 74662**	**CM2**	Antarctica	1				used for model
**Aguas Zarcas**	**CM2**	Fall	0				Predicted
**Bells**	**C2-ung**	Fall	0		1.9 - 2.3	TI (?)	Predicted
**Kolang**	**CM1/2**	Fall	0				Predicted
**Kivesvaara**	**CM2**	Fall	0				Predicted
**Maribo**	**CM2**	Fall	0				Predicted
**Mighei**	**CM2**	Fall	0	2.3	1.6		Predicted
**Murray**	**CM2**	Fall	0	2.4	1.5		Predicted
**Nawapali**	**CM2**	Fall	0				Predicted
**NWA 10834**	**CM1/2**	Hot desert	1				Predicted
**Tarda**	**C2-ung**	Fall	0				Predicted
**Pollen**	**CM2**	Fall	0	2.4			Predicted

spectrum	sample	Figure	NS	AQ [ms]	D1 [ms]	NE	WDW1	WDW2	PR1	PR2
^{1}H NMR	A0106		5616	5000	5000	-	EM	-	1	-
^{1}H NMR	Murchison A5			5000	5000	-	EM	-	1	-
^{1}H, ^{1}H JRES	A0106		1280	1000	500	43	S	S	0	0
^{1}H, ^{1}H JRES	Murchison A5		2048	1000	500	43	Q	Q	0	0
^{1}H, ^{1}H TOCSY	A0106		144	1861	889	1589	EM	Q	0.5	4
^{1}H, ^{1}H TOCSY	Murchison A5		160	1000	500	2243	EM	Q	2.5	2.5

Table S2. Acquisition parameters of NMR spectra, shown according to all figures in the manuscript (Figures 2, S8a-d, S9a-b). NS: number of scans (for 2D NMR: F2); AQ: acquisition time [ms]; D1: relaxation delay [ms]; NE: number of F1 increments in 2D NMR spectra; WDW1, WDW2: apodization functions in F1/ F2 (EM/GM: line broadening factor [Hz]; QS: shifted square sine bell; SI: sine bell); PR1, PR2: coefficients used for windowing functions WDW1, WDW2, EM/GM are given in [Hz], SI/QS derived functions indicate shift by π/n.

δ(^{1}H) [ppm]	key substructures	Hayabusa A0106	Murchison
7.0-10.0 ppm	$C_{ar}\underline{\mathbf{H}}$	2.6	3.8
5.0-7.0 ppm	$=C\underline{\mathbf{H}}$	2.7	0.5
3.1-5.0 ppm	$OC\underline{\mathbf{H}}$	9.2	7.4
2.1-3.1 ppm	$OCC\underline{\mathbf{H}}$	7.7	13.0
1.9-2.1 ppm	acetyl derivatives	6.9	6.8
1.35-1.9 ppm	alkyl	14.2	20.4
1.25-1.35 ppm	$(C\underline{\mathbf{H}}_2)_n$, alkyl	33.7	17.8
1-1.25 ppm	alkyl, $OCC\underline{\mathbf{H}}_3$	8.6	12.1
0.5-1.0 ppm	$CC\underline{\mathbf{H}}_3$	14.5	18.2
0.5-1.9 ppm	sum of $CC\mathbf{H}_n$	77.9	75.3
2.1-5.0 ppm	sum of $OCC\underline{\mathbf{H}}$ + $OC\underline{\mathbf{H}}$	16.9	20.4

Table S3. ^{1}H NMR section integrals (800 MHz, CD_3OD) of methanol A0106 and Murchison extracts (cf. Fig. S2X)

5

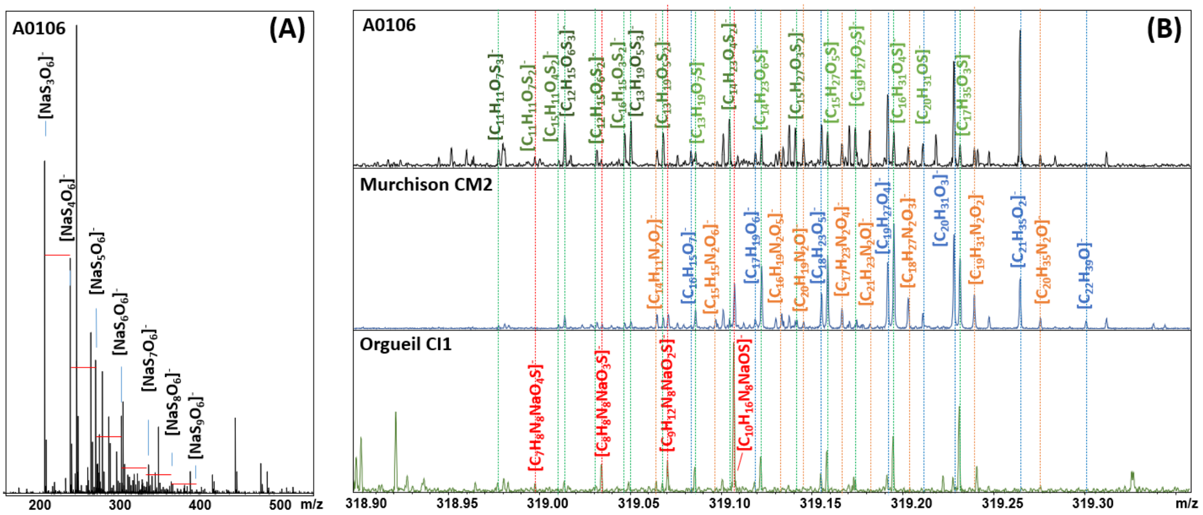

Figure S1: Fourier transform ion cyclotron mass spectrometry of the methanol extract of A0106. (A) full spectra showing the high abundance of polythiols from S3 to S9. (B) details of nominal mass 319 of the same extract compared to the methanol extract of Murchison CM2 and Orgueil CI1 with corresponding annotations in elementary compositions in CHNOSNa. The regular patterns and mass defects reflects homologous compositional series with increasing carbon saturation and lower oxygen contents from left to right (CH_4/O substitution as described in *(17))* (colors are CHO: blue, CHOS: green, CHON: orange, CHONS: red). In A0106 the two annotations on the right, the $C_{17}H_{35}O_3S$ formula being the last in a series of decreasing oxygen number indicates the presence of a sulfonation / -SO_3 compared to $C_{20}H_{31}OS$ in which sulfur rather would be involved as a reduced –SH functional group.

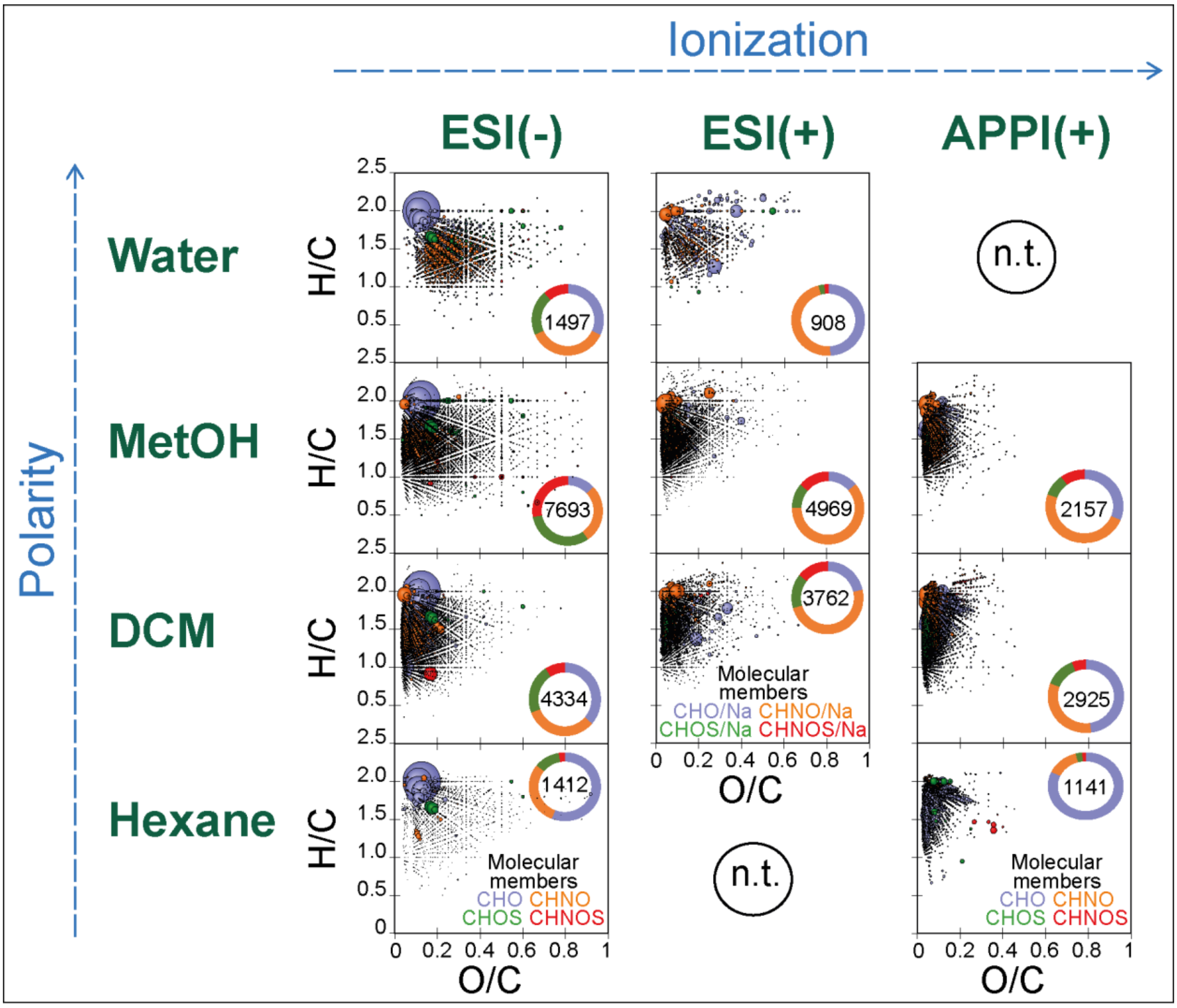

Figure S2: van Krevelen Diagrams of all annotated formula as obtained from ESI(-), ESI+) and APPI(+) ionization modes in FTICR-MS for all available extracts (n.t. not shown the spectra of the hexane extract in ESI(+) mode contained too many impurities and was thus fully non-considered). The bubble size express the intensity in the mass spectra and the color correspond to the chemical families as in the legends. The number of elementary compositions is also shown in each analyzed fraction with the relative abundance in the CHO, CHNO, CHOS and CHNOS chemical families. One clearly observe the increase in oxygenation with increasing polarity of the used solvent. Also APPI enables the profiling of the low oxygenated compounds preferentially.

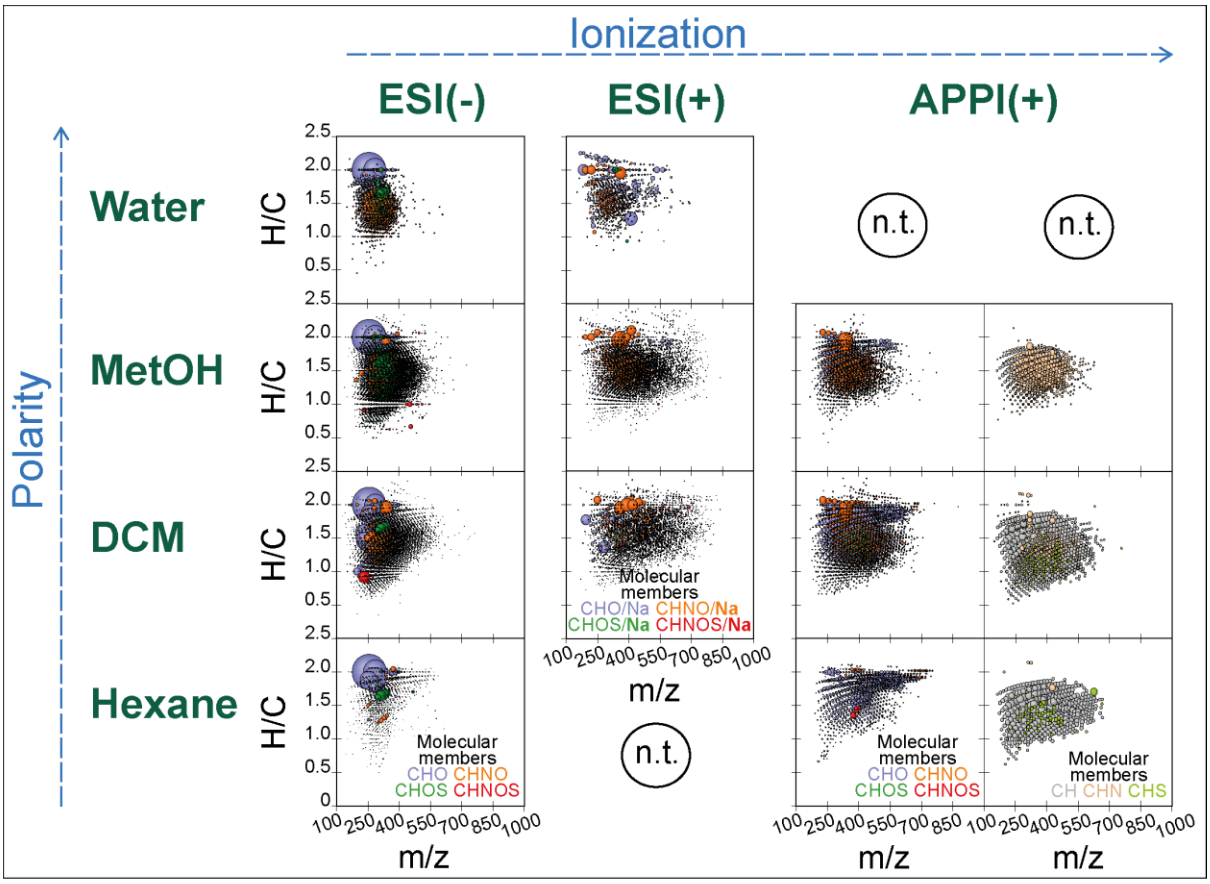

Figure S3: mass edited H/C ratios van Krevelen Diagrams representations of all annotated formula as obtained from ESI(-), ESI+) and APPI(+) ionization modes in FTICR-MS for all available extracts as shown in Figure S2 (n.t. not shown the spectra of the hexane extract in ESI(+) mode contained too many impurities and was thus fully non-considered). One clearly observes the increase in m/z with lower polarity of the used solvent. Also APPI enables the profiling of the higher m/z molecules, thus with lower oxidation states as seen in Figure S2.

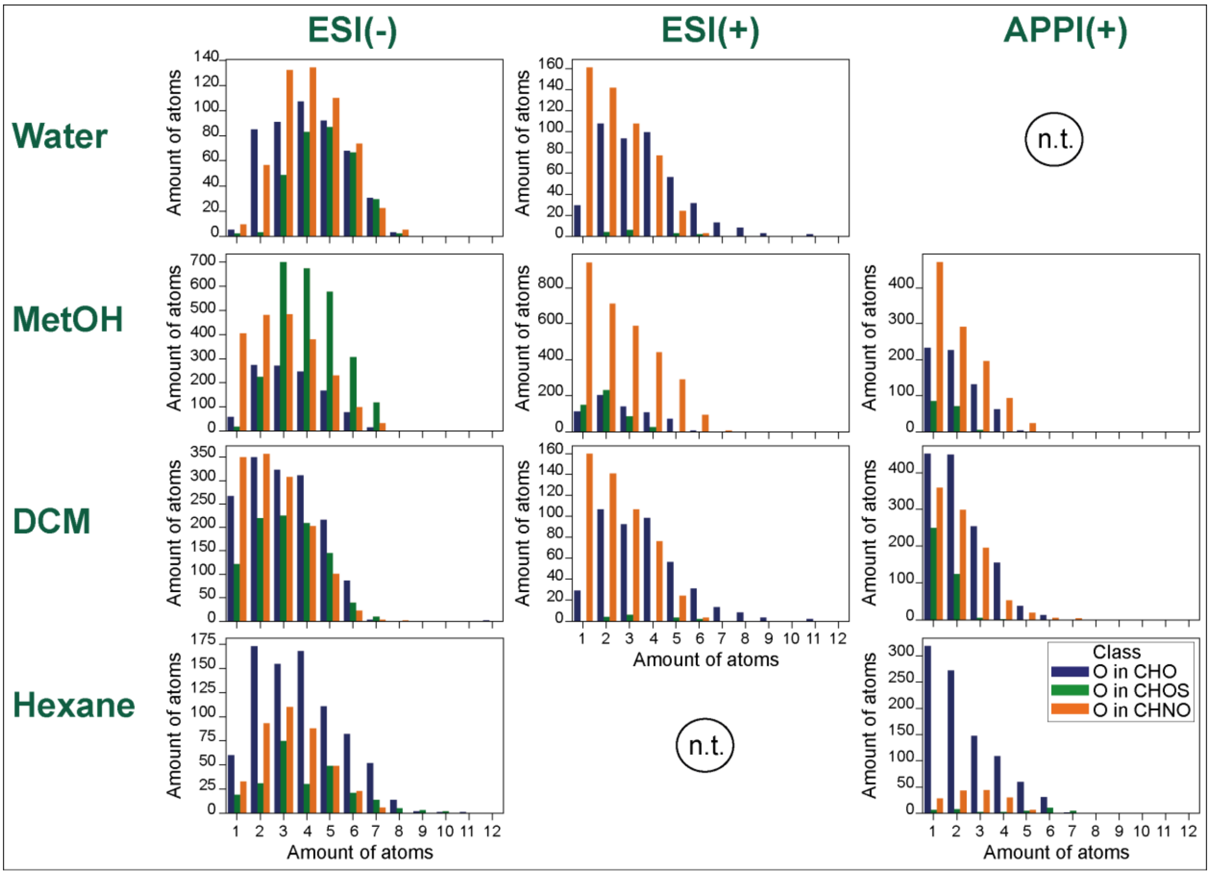

Figure S4: Abundance profiles of the oxygen degree in the formula of the CHO, CHOS and CHNO chemical families as dependent of the extraction solvent and the ionization mode (same data as in Figure S2 and S3). (n.t. not shown the spectra of the hexane extract in ESI(+) mode contained too many impurities and was thus fully non-considered)

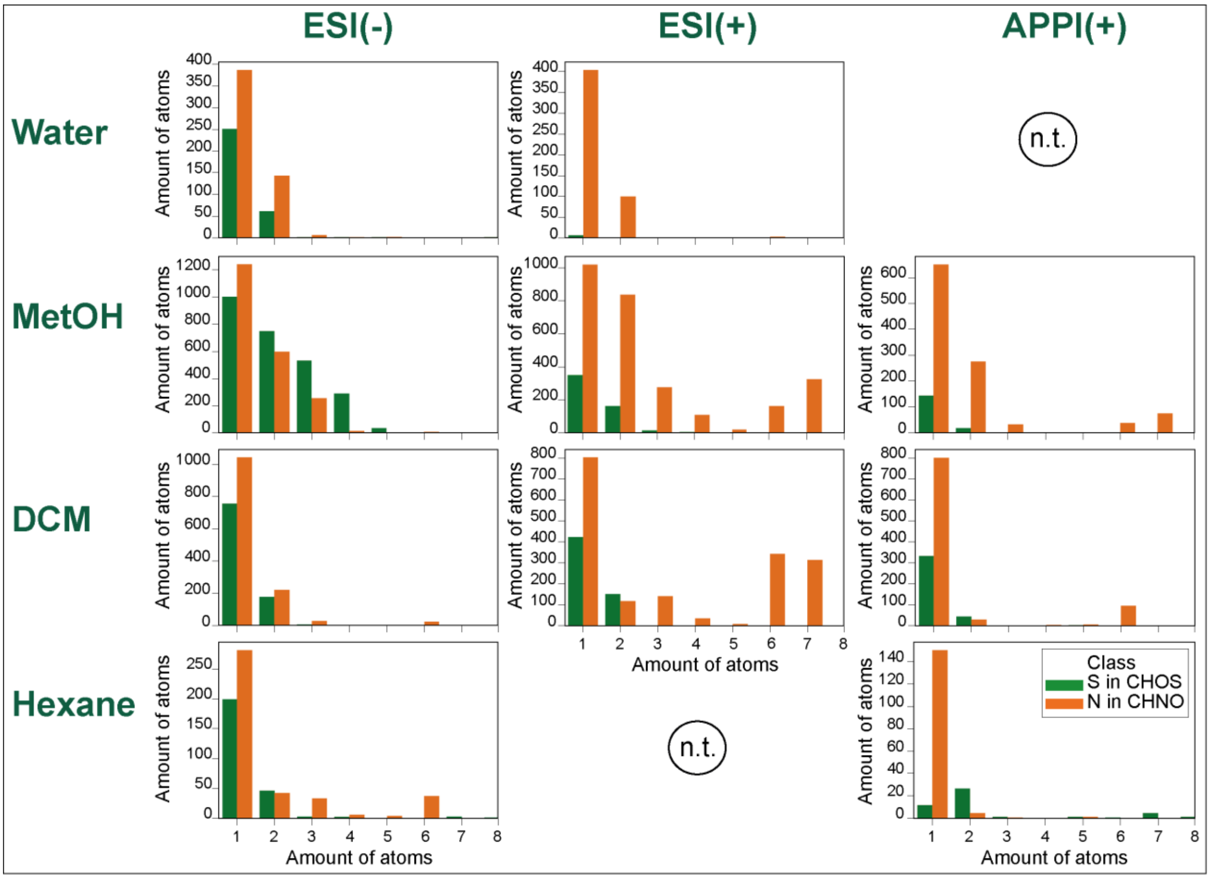

Figure S5: Abundance profiles of the numbers of heteroatoms (S and N) in the formula of the CHOS and CHNO chemical families as dependent of the extraction solvent and the ionization mode (same data as in Figure S2, S3 and S4).

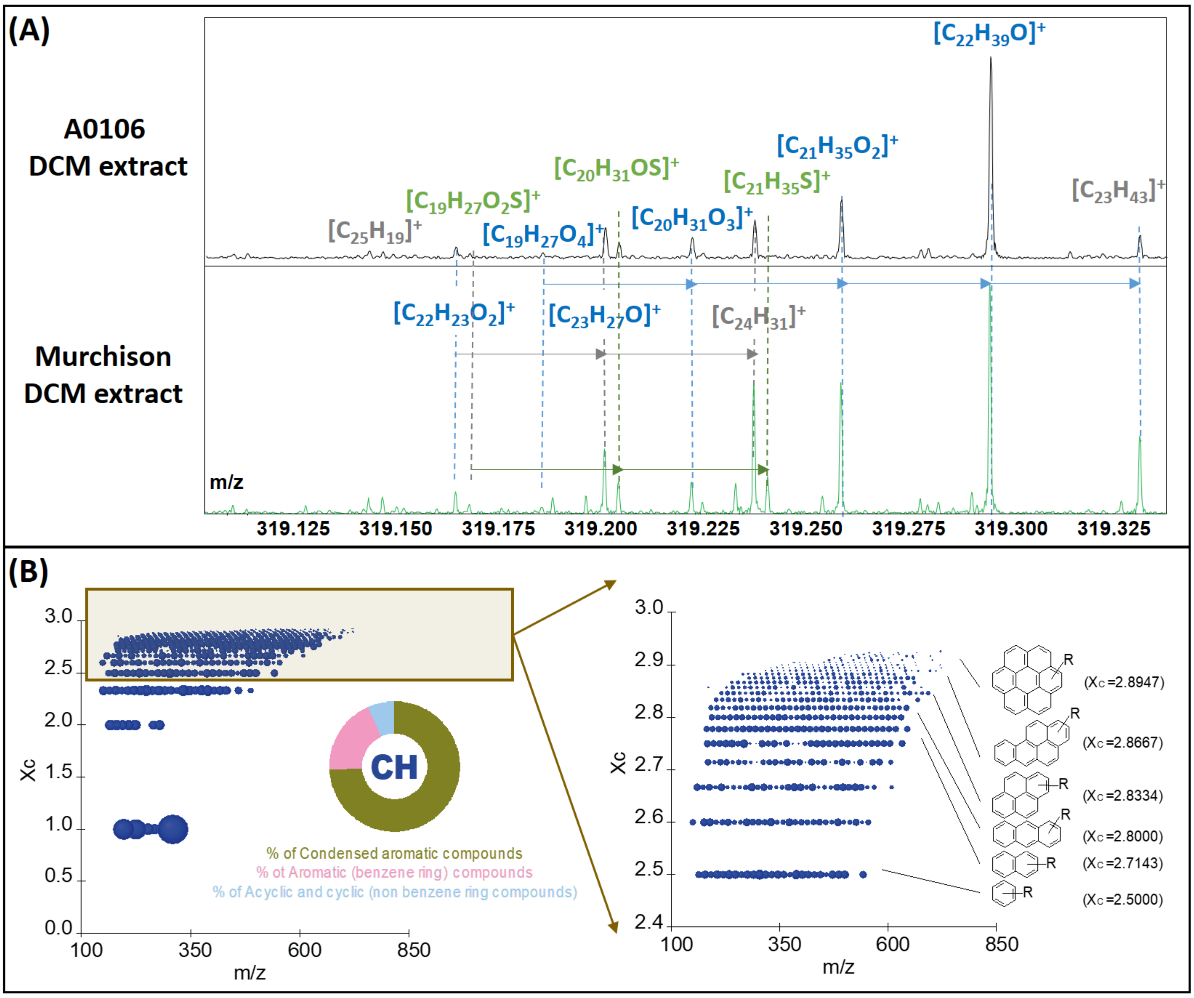

Figure S6: **(A)** Spectra details on nominal mass 319 of the A0106 and Murchison DCM-extracts analyzed in APPi showing similarities in signals with annotated formula and the more diverse molecular profile of Murchison; (B) shows the calculated Aromaticity Equivalent Xc for all CH-type of compounds. *(39)*

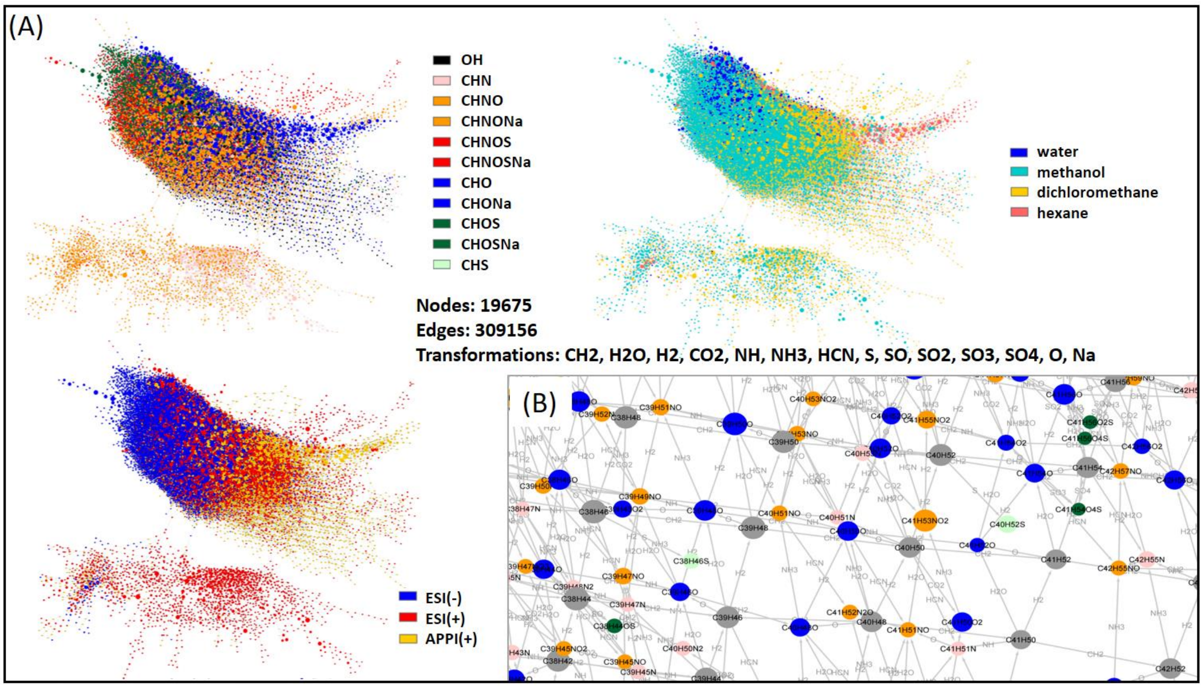

Figure S7 (A) functional network considering the most abundant mass transformations as expressed in Figure 1E and visualized in different color codes enabling the differentiation of the chemical families versus the extraction solvent and the ionization modes, **(B)** Functional connectivity network connecting the experimental elementary compositions (nodes) with possible functional differences such as hydration (H_2O), hydrogenation (H_2), methylation (CH_2), Ammonia insertion (amination+reduction) NH_3, addition of Hydrogen Cyanide HCN.

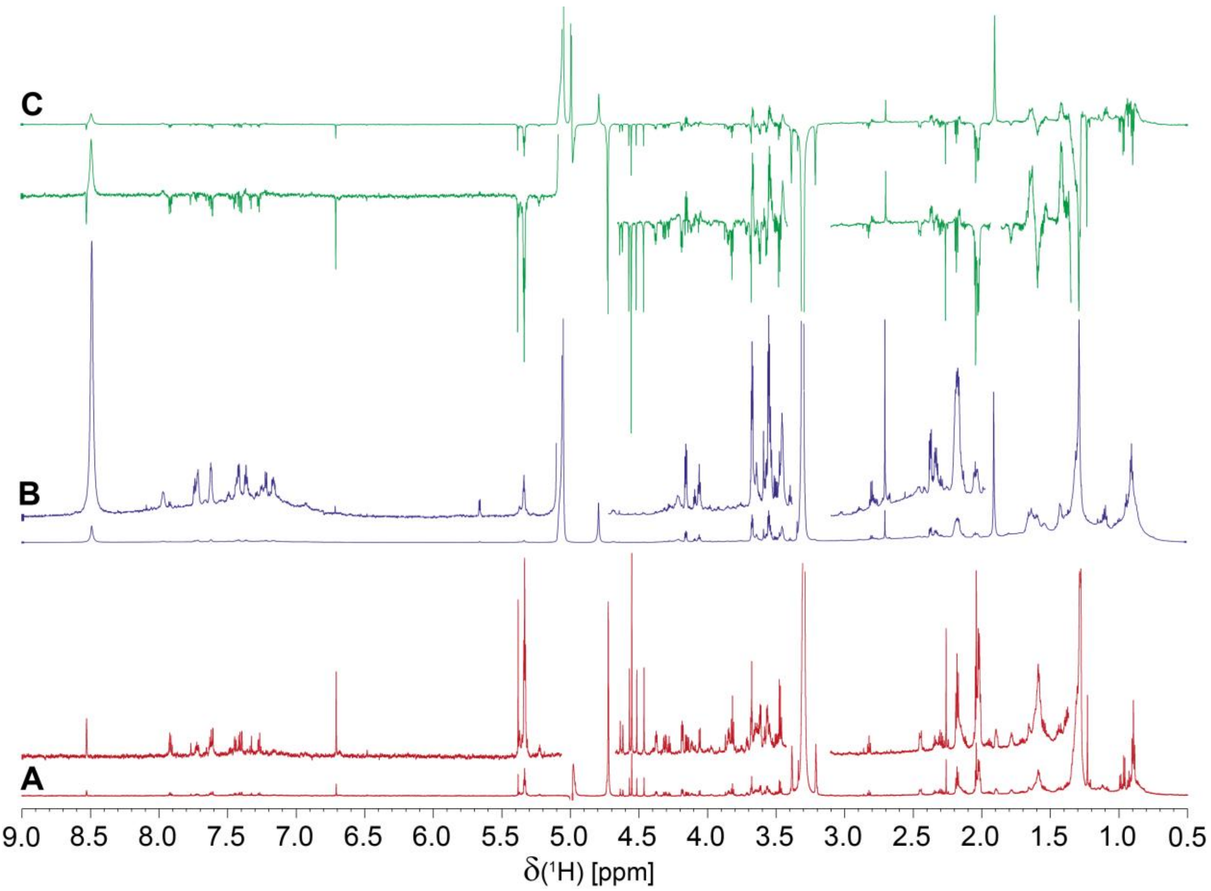

Fig. S8a. ^{1}H NMR spectra (800 MHz, CD_3OD) of methanol extracts of (**A**) A0106, (**B**) Murchison (A5), and (**C**) difference spectra Murchison – A0106, whole range of chemical shift δ_H. Larger linewidth in Murchison extract indicated an overall higher diversity of atomic environments, with special emphasis on remote oxygenation (OC_nC**H** units; n: 1, 2), together with contributions from faster transverse (T_2) relaxation, possibly reflecting enhanced molecular interactions from carboxylic-rich molecules. Smaller linewidths increase ^{1}H NMR resonance amplitude at equal integral. Murchison showed higher relative abundance of pure and remotely oxygenated aliphatic molecules whereas A0106 showed larger proportion of oxygenated aliphatic units (OC**H**) and of oxygenated aromatic molecules with electron-donating OR and electron withdrawing carboxylic COOH substituents.

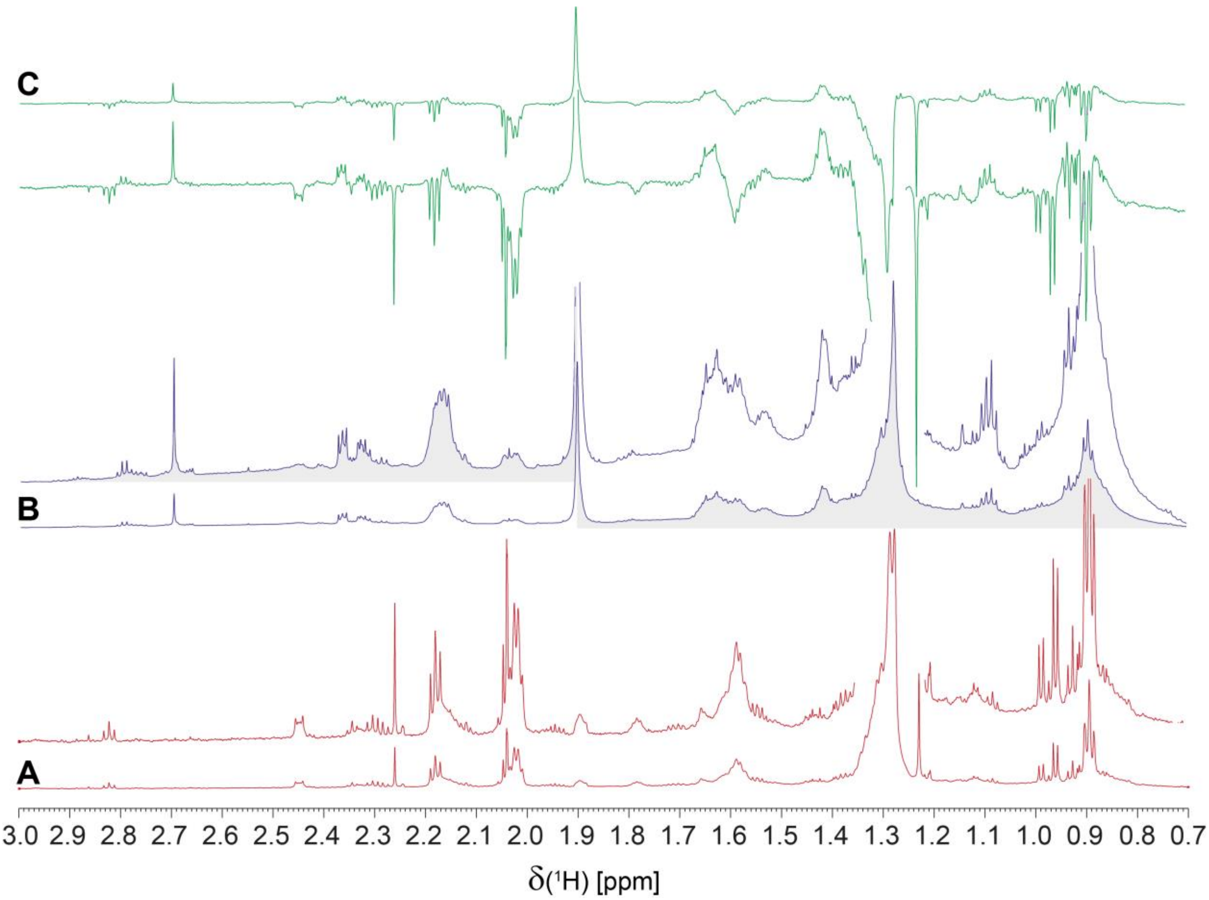

Fig. S8b. ^{1}H NMR spectra (800 MHz, CD_3OD) of methanol extracts of (**A**) A0106, (**B**) Murchison (A5), and (**C**) difference spectra of Murchison – A0106, section of remotely oxygenated (OCC**H** units; δ_H > 1.9 ppm) and pure aliphatic (CCC**H** units; δ_H < 1.9 ppm). ^{1}H NMR resonances of A0106 methanol extract are much narrower than those of Murchison A5. Murchison showed higher relative abundance and diversity of pure and remotely oxygenated aliphatic atomic environments. A0106 extract showed higher relative abundance of a few dozens of relatively abundant (~1% of overall ^{1}H NMR integral) aliphatic atomic environments, which likely comprise small aliphatic carboxylic acids, with isopropyl groups $(H_3C)_2C$**H**-C or related units (H_3C)-C**H**-C_2 (δ_H <~0.9-1.0 ppm).

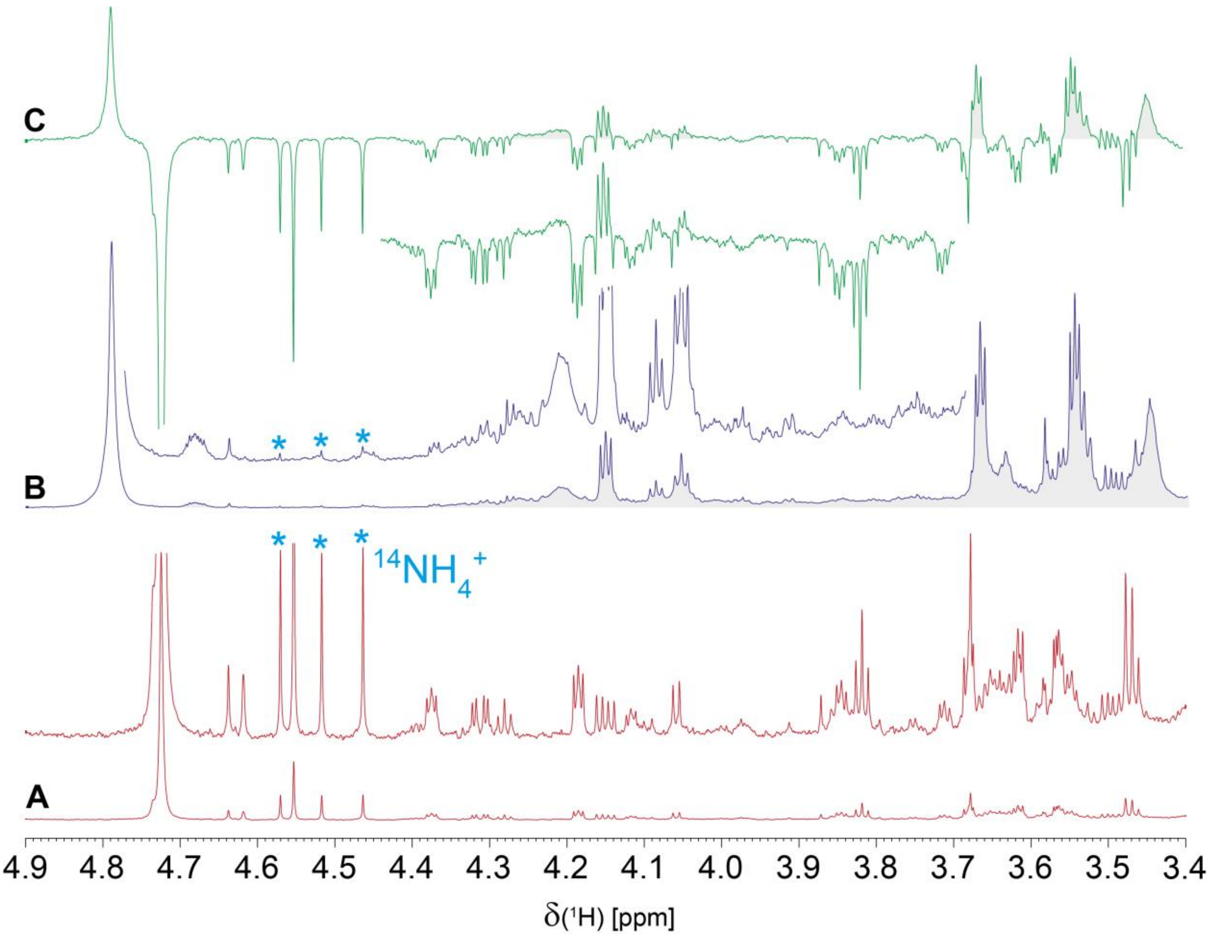

Fig. S8c. ^{1}H NMR spectra (800 MHz, CD_3OD) of methanol extracts of (**A**) A0106, (**B**) Murchison (A5), and (**C**) difference spectra of Murchison - A0106, section of directly oxygenated (OC**H** units). These are more abundant and molecularly diverse in A0106 than those of Murchison extract. ^{1}H NMR resonances of A0106 extract are considerably more narrow than those of Murchison. Ammonium $^{14}NH_4^+$ shows a 1:1:1 triplet ($^{1}J_{NH}$ ~ 42 Hz, with ~1.2 % relative abundance in A0106 , and just visible traces in Murchison.

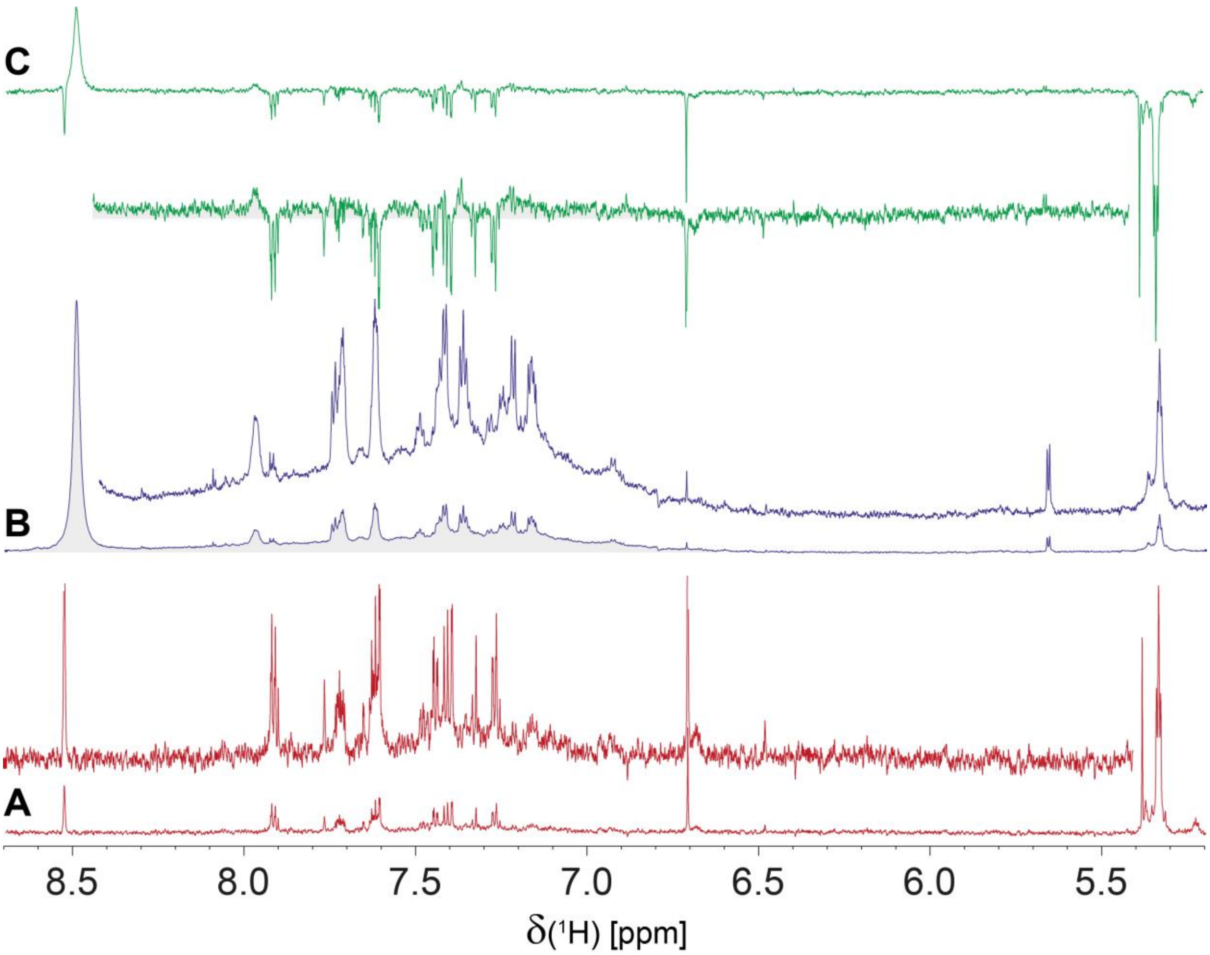

Fig. S8d. ^{1}H NMR spectra (800 MHz, CD_3OD) of methanol extracts of (**A**) A0106, (**B**) Murchison (A5), and (**C**) difference spectra of Murchison – A0106, section of olefinic (C=C**<u>H</u>** units; δ_H < 6 ppm; C_2C=C**<u>H</u>**-CH_2-, δ_H ~5.33 ppm, $^{3}J_{HH}$: 7.3 Hz) and aromatic (C_{ar}**<u>H</u>** units; δ_H > 6 ppm). Major aromatic ^{1}H NMR resonances of both extracts showed gross concordance of δ_H with some variance in relative abundance. Murchison A5 extract *(19)* shows considerably higher proportions of high field ^{1}H NMR resonances (δ_H < 7 ppm) possibly reflecting naphthenic acids with optional admixture of electron-donating oxygen substitution. Low field resonances (δ_H > 7.3 ppm) probably comprise substituted 3-4 units polyaromatic systems and carboxylated benzene derivatives and nitrogen heterocycles.

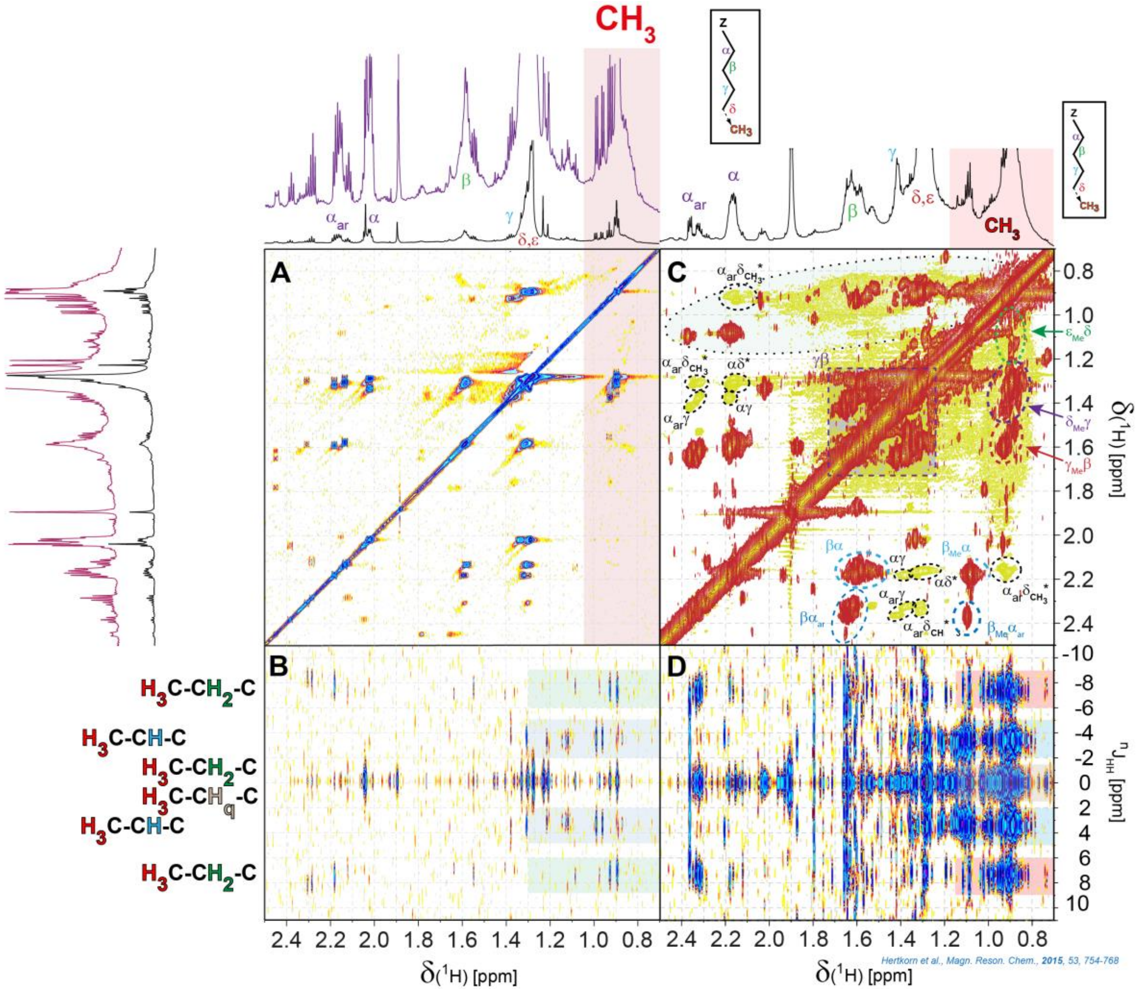

Fig. S9a: Homonuclear 2D NMR spectra (800 MHz, CD_3OD) of Murchison methanol extracts, aliphatic section of OCC**H** (δ_H > 1.9 ppm) and CCC**H** units (δ_H < 1.9 ppm) of A0106 (left **A**, **B**) and and Murchison A5 (right **C**, **D** *(19)*) methanolic extracts; (**A**, **C**) ^1H, ^1H TOCSY NMR spectra (**C**: green shade) and (**C**) ^1H, ^1H COSY NMR spectrum (**C**: red shade), and (**B**, **D**) ^1H, ^1H JRES NMR spectra.

Connectivity networks in A0106 and Murchison extract were largely defined by aliphatic branching and proximity to carboxylic groups and produced a superficial similarity in cross peak positioning of OCC**H** and CCC**H** units in TOCSY and JRES NMR spectra of both meteorite extracts; lower cross peak amplitude was caused by sample limitation in case of A0106. Branched aliphatic molecules in an astrochemical context can be produced by (more) random addition of carbon radical species, and consecutive hydrothermal alteration facilitates further isomerization from linear to branched alkyl motifs because of the higher thermodynamic stability of branched alkyls over linear ones. Specific values of δ_H applied to HOOC-CHα-CHβ-CHγ-CHδ,ε units, and methyl groups. JRES NMR spectra indicated higher proportions of $(H_3C)_2CH$- (doublet splitting; δ_H ~ 0.75-0.96 ppm) than H_3C-CH_2- groups (triplet splitting; δ_H ~ 0.85-0.95 ppm) corroborating presence of methyl-rich compact highly branched alkyl binding motifs. 800 MHz homonuclear 2D NMR spectra with cryogenic detection are very sensitive, and produce significant cross peaks even for very minor constituents of A0106 methanol extracts further contributing to the observed superficial resemblance of major cross peak positions.

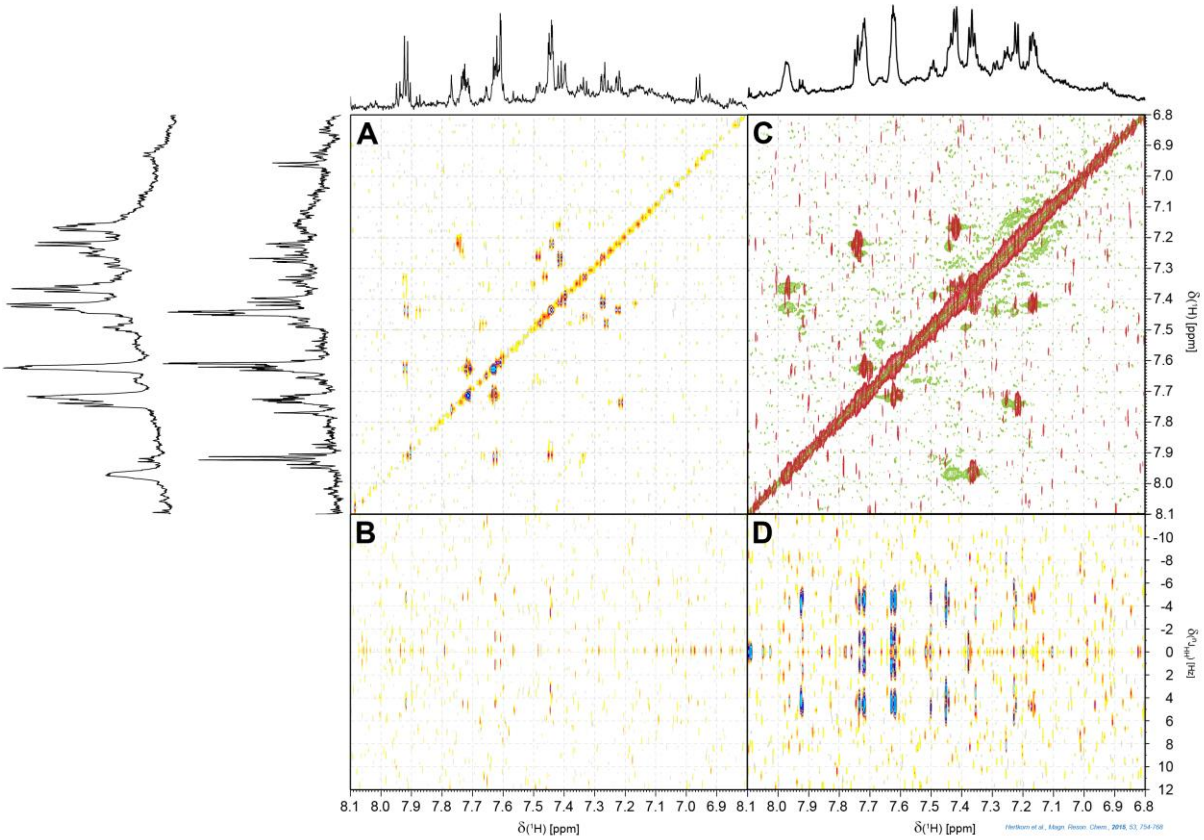

Fig. S9b: Homonuclear 2D NMR spectra (800 MHz, CD_3OD), aliphatic section of aromatic C_{ar}**H** and olefinic =C**H** units of A0106 (left, **A**, **B**) and Murchison (right **C-D)** methanolic extracts; (**A**, **C**) ^{1}H, ^{1}H TOCSY NMR spectra (**C**: green shade) and (**C**) ^{1}H, ^{1}H COSY NMR spectrum (**C**: red shade), and (**B**, **D**) ^{1}H, ^{1}H JRES NMR spectra. A0106 and Murchison methanolic extracts share several of the major aromatic ^{1}H NMR resonances and cross peaks in 2D NMR NMR spectra which likely represent polyaromatic rings. Owing to limited sample, mDHOM showed only a very few cross peaks whereas Murchison extract showed background cross peaks of benzene derivatives with alkyl, carboxyl, and oxygen functionalization (*19*).

***The Hayabusa2-initial-analysis SOM team:** Philippe Schmitt-Kopplin[1,2,3], Norbert Hertkorn[1], Hiroshi Naraoka[7], Yoshinori Takano[5], Jason P. Dworkin[6], Kenji Hamase[15], Aogu Furusho[15], Minako Hashiguchi[14], Kazuhiko Fukushima[16], Dan Aoki[16], José C. Aponte[6], Eric T. Parker[6], Daniel P. Glavin[6], Hannah L. McLain[6], Jamie E. Elsila[6], Heather V. Graham[6], John M. Eiler[17], Alexander Ruf[18], Francois-Regis Orthous-Daunay[4], Junko Isa[19], Véronique Vuitton[4], Roland Thissen[20], Nanako O. Ogawa[21], Saburo Sakai[21], Toshihiro Yoshimura[21], Toshiki Koga[21], Haruna Sugahara[9], Naohiko Ohkouchi[21], Hajime Mita[22], Yoshihiro Furukawa[10], Yasuhiro Oba[23], Shogo Tachibana[8,9].*

Philippe Schmitt-Kopplin, schmitt-kopplin@tum.de
[1]Helmholtz Munich, Analytical BioGeoChemistry; Ingolstaedter Landstraße 1, 85764 Neuherberg, Germany.
2Technische Universität München, Analytische Lebensmittel Chemie; Maximus-von-Forum 2, 85354 Freising, Germany.
[3]Max Planck Institute for Extraterrestrial Physics; Gießebachstraße 1, 85748 Garching bei München, Germany.
schmitt-kopplin@tum.de

Norbert Hertkorn, n.hertkorn@gmx.de
[1]Helmholtz Munich, Analytical BioGeoChemistry; Ingolstaedter Landstraße 1, 85764 Neuherberg, Germany.

Hiroshi Naraoka, naraoka@geo.kyushu-u.ac.jp
[7]Department of Earth and Planetary Sciences, Kyushu University; Motooka 744, Nishiku, Fukuoka, 819-0395, Japan,

Yoshinori Takano, takano@jamstec.go.jp
[5]Biogeochemistry Research Center (BGC), Japan Agency for Marine-Earth Science and Technology (JAMSTEC), 2-15 Natsushima, Yokosuka 237-0061, Japan.

Jason P. Dworkin, jason.p.dworkin@nasa.gov
[6]Solar System Exploration Division, NASA Goddard Space Flight Center, Greenbelt, Maryland 20771, USA.

Kenji Hamase, hamase@phar.kyushu-u.ac.jp
[15]Graduate School of Pharmaceutical Sciences, Kyushu University, Motooka 744, Nishi-ku, Fukuoka 819-0395, Japan.

Aogu Furusho, furusho@u-shizuoka-ken.ac.jp
[15]Graduate School of Pharmaceutical Sciences, Kyushu University, Motooka 744, Nishi-ku, Fukuoka 819-0395, Japan.;

Minako Hashiguchi, hashiguchi@eps.nagoya-u.ac.jp
[14]Graduate School of Environmental Studies, Nagoya University, Furo-cho, Chikusa-ku, Nagoya, 464-8601, Japan

Kazuhiko Fukushima, Kazuhiko Fukushima, kazu@agr.nagoya-u.ac.jp
[16]Graduate School of Bioagricultural Sciences, Nagoya University; Chigusa-ku, Nagoya, 464-8601, Japan

Dan Aoki, daoki@agr.nagoya-u.ac.jp
[16]Graduate School of Bioagricultural Sciences, Nagoya University; Chigusa-ku, Nagoya, 464-8601, Japan

José C. Aponte, Jose.C.Aponte@nasa.gov
[6]Solar System Exploration Division, NASA Goddard Space Flight Center, Greenbelt, Maryland 20771, USA.

Parker T. Eric, eric.t.parker@nasa.gov
[6]Solar System Exploration Division, NASA Goddard Space Flight Center, Greenbelt, Maryland 20771, USA.

Daniel P. Glavin, daniel.p.glavin@nasa.gov
[6]Solar System Exploration Division, NASA Goddard Space Flight Center, Greenbelt, Maryland 20771, USA.

Hannah L. McLain, hannah.l.mclain@nasa.gov
[6]Solar System Exploration Division, NASA Goddard Space Flight Center, Greenbelt, Maryland 20771, USA.

Jamie E. Elsila, Jamie.Elsila@nasa.gov
[6]Solar System Exploration Division, NASA Goddard Space Flight Center, Greenbelt, Maryland 20771, USA.

Heather V. Graham, heather.v.graham@nasa.gov
[6]Solar System Exploration Division, NASA Goddard Space Flight Center, Greenbelt, Maryland 20771, USA.

John M. Eiler, eiler@gps.caltech.edu
[17]Division of Geological and Planetary Sciences, California Institute of Technology, Pasadena, California 91125, USA.

Alexander Ruf, rufalexan@gmail.com
[18]Excellence Cluster ORIGINS, Garching 85748, Germany.

Francois-Regis Orthous-Daunay, frod@univ-grenoble-alpes.fr
[4]Université Grenoble Alpes, CNRS, CNES, IPAG; 38000 Grenoble, France

Junko Isa, jisa@elsi.jp
[19]Earth-Life Science Institute (ELSI), Tokyo Institute of Technology, Meguro-ku, Tokyo 152-8550, Japan.

Véronique Vuitton, veronique.vuitton@univ-grenoble-alpes.fr
[4]Université Grenoble Alpes, CNRS, CNES, IPAG; 38000 Grenoble, France

Roland Thissen, Roland.thissen@u-psud.fr
[20]Université Paris-Saclay, CNRS, Institut de Chimie Physique, UMR8000, 91405 Orsay, France.

Nanako O. Ogawa, nanaogawa@jamstec.go.jp
[21]Biogeochemistry Research Center (BGC), Japan Agency for Marine-Earth Science and Technology (JAMSTEC), 2-15 Natsushima, Yokosuka 237-0061, Japan.

Saburo Sakai, saburos@jamstec.go.jp
[21]Biogeochemistry Research Center (BGC), Japan Agency for Marine-Earth Science and Technology (JAMSTEC), 2-15 Natsushima, Yokosuka 237-0061, Japan.

Toshihiro Yoshimura, yoshimurat@jamstec.go.jp
[21]Biogeochemistry Research Center (BGC), Japan Agency for Marine-Earth Science and Technology (JAMSTEC), 2-15 Natsushima, Yokosuka 237-0061, Japan.

Toshiki Koga, toshikikoga@jamstec.go.jp
[21]Biogeochemistry Research Center (BGC), Japan Agency for Marine-Earth Science and Technology (JAMSTEC), 2-15 Natsushima, Yokosuka 237-0061, Japan.

Haruna Sugahara, sugahara.haruna@jaxa.jp
[9]Institute of Space and Astronautical Science, Japan Aerospace Exploration Agency (ISAS/JAXA), Sagamihara 252-5210, Japan.

Naohiko Ohkouchi, nohkouchi@jamstec.go.jp
[21]Biogeochemistry Research Center (BGC), Japan Agency for Marine-Earth Science and Technology (JAMSTEC), 2-15 Natsushima, Yokosuka 237-0061, Japan.

Hajime Mita, mita@fit.ac.jp
[22]Department of Life, Environment and Applied Chemistry, Fukuoka Institute of Technology, Higashi-ku, Fukuoka 811-0295, Japan.

Yoshihiro Furukawa, furukawa@tohoku.ac.jp
[10]Department of Earth Material Science, Tohoku University, Aoba-ku, Sendai 980-8578, Japan.

Yasuhiro Oba, oba@lowtem.hokudai.ac.jp
[23]Institute of Low Temperature Sciences (ILTS), Hokkaido University, Kita-ku, Sapporo 060-0810, Japan.

Shogo Tachibana, tachi@eps.s.u-tokyo.ac.jp
[8]Tokyo Organization for Planetary and Space Science, University of Tokyo, Bunkyo-ku, Tokyo 113-0033, Japan.
[9]Institute of Space and Astronautical Science, Japan Aerospace Exploration Agency (ISAS/JAXA), Sagamihara 252-5210, Japan.
